# Myocardial Revascularization Surgery in COVID-19: Choosing the Most Opportune Moment for Intervention

**DOI:** 10.21470/1678-9741-2020-0405

**Published:** 2020

**Authors:** Anderson da Rosa Rosado, Franciele Kuhn Mesacasa, Leopoldo Moratelli Neto

**Affiliations:** 1 Instituto de Cardiologia de Santa Catarina - Cirurgia Cardíaca, São José, SC, Brazil. E-mail: leopoldomoratellineto@gmail.com

Dear Editor,

The current pandemic scenario caused by coronavirus disease (COVID-19) has emerged as a global health emergency, affecting almost every country in the world. It is known that, in addition to the involvement of the respiratory system and its highly infectious nature, the presence of underlying cardiovascular disease has more than tripled fatality rate of patients with this comorbidity^[1]^. We present a case report with a positive result not only for the patient and our service, but also to provide hope in the face of the present-day struggle.

Female patient, 55 years old, sought medical attention in the emergency room of the Instituto de Cardiologia de Santa Catarina, showing fatigue, dyspnea on medium exertion, dry cough for seven days with greater intensity at bedtime. On physical examination, the patient showed a good general condition, hydrated, a symmetrical vesicular murmur present, bibasilar crackles, normal heart sounds and regular rhythm, with no murmurs. An electrocardiogram was performed in the emergency room, showing a low-voltage QRS complex, an electrically inactive lower zone with positive ultrasensitive troponin enzyme in the first 12 hours--sequential values: 888 ng/mL, 1460 ng/mL and 1798 ng/mL. The following diagnostic hypotheses were discussed: heart failure and acute myocardial infarction that evolved from the lower wall.

**Fig. 1:**
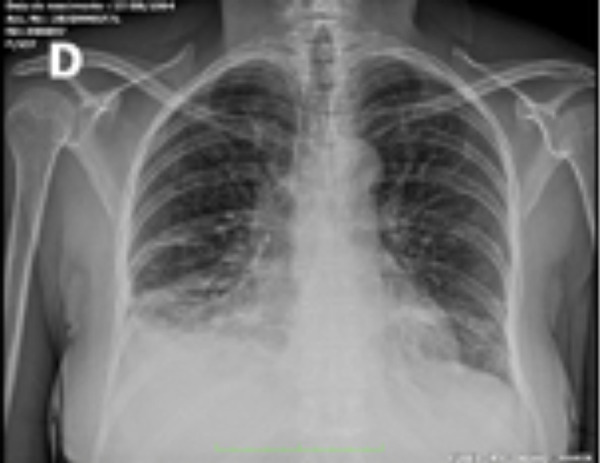
Anteroposterior chest radiograph.

**Fig. 2:**
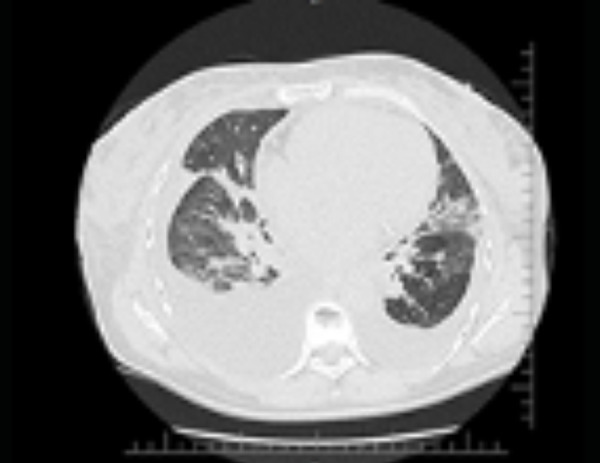
Chest tomography.

Due to the pandemic, all patients screened with suspected SARS-COVID-2 infection are placed in respiratory isolation and undergo serology test with RT-PCR (reverse transcription polymerase chain reaction). In the evaluation of laboratory tests, arterial blood gases with hypoxemia and respiratory acidosis (pH 7.36, pO_2_ 58.4, pCO_2_, 39.8, BE -3, sO_2_ 89%) were found, as well as D-dimer of 1580 ng/mL, eosinophilia (69 mm^3^) and normal lymphocytes. Chest radiography (Figure 1): thickening of the pleuropulmonary interstitium, infiltration of the alveolar pattern in the right lower lobe, obliteration of the right costophrenic sinus. On chest tomography (Figure 2): bilateral pleural effusion, ground-glass opacities and smooth thickening of the sparse interlobular septa throughout the lungs. Consolidation of air space with air bronchograms in the lower lobes. The RT-PCR test was positive.

Hospitalization for intravenous antibiotic therapy with ceftriaxone 1 g twice a day and azithromycin 500 mg once a day; started clopidogrel 75 mg and acetylsalicylic acid 100 mg once a day. Among the diagnoses already mentioned, the new hypothesis is SARS-COVID-2 infection evolving to pneumonia. The patient remained hemodynamically stable, tolerating oxygen therapy via a nasal catheter, with clinical improvement of dyspnea and blood gas analysis. The patient did not evolve to orotracheal intubation.

**Fig. 3:**
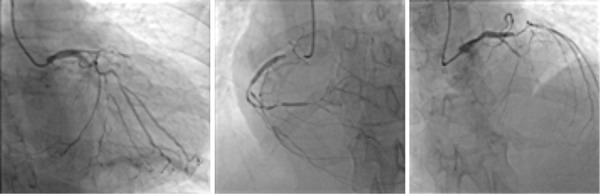
Cardiac catheterization. (A) right coronary lesion 90% proximal, 70% distal. (B) Circumflex artery lesion 70% distal and marginal artery with lesion 80% proximal. (C) main left coronary artery without changes, descending coronary with total collusion.

Cardiac catheterization (Figure 3) was performed for stratification of coronary disease, which showed triple vessel coronary disease with indication for surgical treatment. Cardiovascular nuclear magnetic resonance (NMR) imaging showed signs of large myocardial ischemia, LVEF 33% (Simpson) and viral myocarditis was ruled out.

We chose to wait 20 days after the diagnosis of COVID-19, waiting for the end of treatment with antibiotics for 14 days for bacterial pneumonia, according to the institution's Hospital Infection Control Committee protocol, before subsequently performing cardiac surgery. However, with no improvement in the white blood cell count, which on the day before surgery presented significant leukopenia and eosinophilia (leukocytes: 2,810 cells/mm^3^; eosinophils: 89.92 cells/mm^3^), the patient's clinical stability and the post-infarction time were taken into consideration to decide the ideal time for surgical approach. The unavailability of a new test for COVID-19 at the institution at the time meant that strict adherence to the droplet contact prevention protocol was carefully adopted in the surgery to protect the team.

The coronary artery bypass graft surgery with cardiopulmonary bypass was performed on 05/05/2020, with the proposal for revascularization of the internal thoracic artery to the anterior descending coronary and left internal saphenous vein to the circumflex coronary in its marginal branch. She was transferred to a coronary care unit and was extubated in less than 6 hours. She was discharged on the second postoperative day to the ward, remaining hemodynamically stable, with no electrical changes in the electrocardiogram or neurological status. Hospital discharge occurred seven days post-surgery.

At the time of a pandemic, in which a disease that is still little-known plagues us, the uncertainty of the best time to perform the indicated treatment is a challenge for the medical team. In our institution, we take into account the patient's clinical status and the choice of early intervention is due to the fact that studies show that, in patients with non-ST elevation acute coronary syndrome, there was a significant improvement after 6 months, in the quality of life of those who underwent revascularization in early years, basically at the expense of decreasing angina episodes^[2]^. In addition, the presentation of positive results, in addition to hope, brings us the certainty that even in the face of serious conditions like these, the evolution of medicine and the careful treatment of the patient can have a successful result.
